# Risk for Severe Intimate Partner Violence in Nairobi’s Informal Settlements: Tailoring the Danger Assessment to Kenya

**DOI:** 10.9745/GHSP-D-23-00116

**Published:** 2024-02-28

**Authors:** Shannon N. Wood, Nancy Perrin, Irene Akumu, Ben Asira, Amber Clough, Nancy Glass, Jacquelyn Campbell, Michele R. Decker

**Affiliations:** a Department of Population, Family, and Reproductive Health, Johns Hopkins Bloomberg School of Public Health, Baltimore, MD, USA.; b Johns Hopkins School of Nursing, Baltimore, MD, USA.; c Mashinani Department, Ujamaa-Africa, Nairobi, Kenya.; d Department of International Health, Johns Hopkins Bloomberg School of Public Health, Baltimore, MD, USA.; e Center for Global Health, Johns Hopkins University, Baltimore, MD, USA.; f Department of Health Policy and Management, Johns Hopkins Bloomberg School of Public Health, Baltimore, MD, USA.; g Center for Public Health and Human Rights, Johns Hopkins Bloomberg School of Public Health, Baltimore, MD, USA.

## Abstract

**Introduction:**

Understanding the risk for severe intimate partner violence (IPV) can help women and providers assess danger. The validated, widely used Danger Assessment (DA) developed for this purpose has not been tested in a low- and middle-income country (LMIC). We tailored the DA to Nairobi, Kenya, and prospectively evaluated baseline danger against severe IPV at 3-month follow-up.

**Methods:**

We used data from the myPlan Kenya trial conducted in 3 informal settlements in Nairobi, Kenya, from 2017 to 2018. DA items were refined through formative and pilot phases, yielding minor wording modifications. Quantitative analyses prospectively evaluated baseline DA against severe IPV at 3-month follow-up to understand the predictive effect of the (1) original 20-item DA, (2) 16-item Kenya-DA (highest relative risk ratios [RRR] with severe IPV), and (3) 16-item Kenya-DA weighted (weighting based on strength of RRRs). Diagnostic criteria, including C-statistics, sensitivity, specificity, receiver operating characteristic curve, and area under the curve, were examined; logistic regressions quantified the odds of each metric predicting severe IPV at follow-up.

**Results:**

The original 20-item DA produced the highest specificity (75.41%) and lowest sensitivity (57.14%), resulting in the overall lowest C-statistic. Compared to the 16-item Kenya-DA, the Kenya-DA weighted produced slightly higher sensitivity (66.67% vs. 64.29%) and specificity (77.05% vs. 72.13%), resulting in the highest C-statistic (0.78 vs. 0.75). All versions successfully predicted severe IPV at 3-month follow-up (original DA: odds ratio [OR]=1.26, 95% confidence interval [CI]=1.12, 1.41, *P*<.001; Kenya-DA: OR=1.33, 95% CI=1.16, 1.53, *P*<.001; Kenya-DA weighted: OR=1.19, 95% CI=1.10, 1.28, *P*<.001). Several factors identified as homicide risk factors in other settings were not prospectively associated with severe IPV.

**Conclusion:**

Within a high-danger LMIC context, all 3 DA configurations performed well diagnostically. We recommend the 16-item Kenya-DA given the value for simplicity and field implementation, whereas the Kenya-DA weighted can add accuracy for research purposes.

## Abstract (precis)

Although a tailored 16-item weighted danger assessment may be valuable for research purposes, the unweighted 16-item Kenya-Danger Assessment is most valuable for implementation among practitioners working directly with intimate partner violence survivors, given simplicity for field implementation.

## Teaser

**Key Findings:**

- Two original Danger Assessment (DA) items were modified to increase interpretation within and relevance for the Nairobi context. The pilot phase further revealed that the messages to women about their DA needed to be altered to reflect the high-danger context.
- We found that within this context, all 3 DA configurations—original DA (inclusive of all 20 items), Kenya-DA (based on items with highest relative risk ratios with severe IPV), and weighted Kenya-DA (based on items with highest relative risk ratios with severe IPV and weighting dependent on the strength of these associations)—performed well diagnostically and predicted severe IPV at 3-month follow-up.

**Key Implications:**

- Practitioners working directly with women experiencing IPV, including health, justice, housing, psychosocial/counseling, and violence support services, should implement the 16-item Kenya DA, given simplicity for field implementation.
- Researchers requiring additional accuracy in assessing danger or homicide risk can opt to use the Kenya-DA weighted.

## INTRODUCTION

Intimate partner violence (IPV), or physical, sexual, or emotional violence perpetrated within an intimate partnership, is pervasive and can be lethal—1 in 3 women globally will experience IPV during the course of their lifetimes,^1^ and one-third of female homicides are perpetrated by an intimate partner.^2^ Moreover, IPV is the single largest risk factor for intimate partner homicide.^3^ Although the adverse health effects of IPV are well documented,^4^^,^^5^ mortality impact and risks thereof are difficult to ascertain given the poor quality of death certificate data, particularly within low- and middle-income countries, and limited reporting of intimate partner relationships.^2^ Given these measurement challenges, escalation of violence (i.e., severe IPV) may serve as a proximal outcome for more lethal abuse.^6^

Risk assessments are important tools to help women facing abuse understand the degree of danger in their violence experiences to make a safety plan to try and prevent the escalation of violence to the point of lethality; these tools also serve as important targeted intervention points for health and justice response providers.^6^^–^^9^ The Danger Assessment (DA), developed by Campbell and colleagues in the United States, is one such assessment that has proved useful in helping women assess their own level of danger from a partner.^10^ Notably, the development of the DA was a large research endeavor requiring extensive resources given its utilization of quantitative homicide data and comparison with other abused women.^10^^–^^13^ Initial creation of the tool included the development of items to assess potential risk behaviors for intimate partner homicide that occurred both before the violent incident (i.e., perpetrator access to a gun, previous threat with weapon, stepchild within the home, and estrangement) and those that occurred during the violent incident (i.e., perpetrator’s use of a gun and having left the partner).^14^ The research team then validated this tool via an 11-city case-control study to assess the predictive validity of risk factors for femicide, revise items and develop a scoring algorithm, and test this revised DA among an independent sample.^10^ Although other assessments have largely focused on the risk of repeat assault and severe IPV, to date, the DA is the most widely used, most valid, and most reliable homicide risk assessment; however, its external validity is unknown beyond the United States.^7^

Tailoring of the DA to other contexts and populations can help violence prevention and response researchers and practitioners understand who is at risk for the most severe violence. Within the United States, this tool has been tailored for female same-sex relationships^15^ and immigrant women^16^; to date, however, such item modifications have not been made to understand lethality risk within low- and middle-income countries, despite the demonstrated IPV homicide risk and continued advocacy efforts to integrate risk assessments into practice settings to link IPV survivors directly to violence resources.^7^ Tailoring processes, similar to those previously undertaken, may be helpful for understanding local risk contexts and contributors to the most severe forms of abuse. Such work is also being undertaken in Brazil, but final evaluation data from that project have not yet been published.^17^

Tailoring of the DA to other contexts and populations can help violence prevention and response researchers and practitioners understand who is at risk for the most severe violence.

In Africa, it is estimated that over 40% of female homicides are perpetrated by an intimate partner.^2^ In many sub-Saharan African countries, past-year IPV prevalence estimates exceed 30%.^1^ Moreover, hotspots (i.e., high-violence areas) may perpetuate risks for intimate partner homicide. Namely, urban informal settlements, including those within Nairobi, Kenya, are widely viewed as high-danger contexts, particularly for violence perpetrated against women and girls.^18^^–^^22^ Given the profound need to prevent IPV homicide globally, this study aimed to tailor the DA to urban Nairobi, Kenya, and sought to understand how U.S.-based homicide risk factors were applicable to IPV survivors within this high-danger setting. As part of the parent study, self-assessed homicide risk factors were embedded into a safety decision aid for women to help women understand the danger they were facing. The objectives of this secondary analysis were to evaluate danger items at baseline against severe IPV at 3-month follow-up to understand the predictive effect of 3 DA configurations: (1) original DA (inclusive of all 20 items); (2) Kenya-DA (based on items with highest relative risk ratios with severe IPV); (3) weighted Kenya-DA (based on items with highest relative risk ratios with severe IPV and weighting dependent on the strength of these associations). The strengths and weaknesses of each DA configuration and utility for violence research and prevention practice are discussed based on prediction and diagnostic testing.

## METHODS

### Overview of Study

Longitudinal data were collected as part of the myPlan Kenya community-based randomized controlled trial (RCT)^23^^,^^24^ conducted in 3 informal settlements in Nairobi, Kenya, in collaboration with Ujamaa-Africa, a violence prevention and response organization. MyPlan Kenya is a web-based safety decision aid for women experiencing IPV.^23^^,^^24^ Study sites of Korogocho/Kariobangi, Dandora, and Huruma/Mathare are densely populated, informal settlement communities with numerous safety hazards for women^18^^,^^19^^,^^21^; sites were selected due to feasibility and community reach for the parent study.

### Formative Phase and Danger Assessment Adaptation

Before the RCT implementation, study procedures and myPlan Kenya app content were refined through: (1) a qualitative formative phase (June–August 2017) consisting of focus group discussions with key informants (N=16) and women experiencing IPV (N=43) to review app mock-ups and discuss content and survey priorities, and (2) a quantitative pilot phase (December 2017; N=18 IPV survivors) to test app flow and key messages. These refinement steps yielded minor wording modifications for the DA items to ensure comprehensibility and relevance for the Nairobi study population, as well as changes to the results screen, which gives the woman the results of her DA assessment once she has completed all survey items.

### Randomized Controlled Trial Procedures

After completion of formative and pilot phases, the RCT began recruitment in March 2018 using community-based methods, including flyers, presentations, and word-of-mouth. Women aged 18–35 years, who had reported physical or sexual IPV or partner-related fears within the past 3 months, resided in study communities with no plans to move in the next 6 months, and spoke English or Swahili were recruited. Eligibility, oral informed consent, and data collection all occurred in person in private offices with trained research assistants. All data collection, including randomization, consent, and administration of intervention and control apps, occurred via tablet; participants were given the option to complete independently or with help from a research assistant.

Upon completion of the baseline survey, all participants were randomized 1:1 to intervention or control arms. Intervention participants received the myPlan Kenya app; control participants received a standard set of referrals to IPV-related legal, health, safety, counseling, and financial resources. The myPlan Kenya app sections comprised: (1) Healthy Relationships—an educational component to define healthy and unhealthy relationships and help women reflect on their own relationship; (2) My Relationship—information on relationship characteristics to inform the tailoring of safety strategies; (3) Red Flags—an assessment of warning signs; (4) My Safety—all items from the original DA to help self-assess risk for severe and lethal violence^10^; (5) My Priorities—pairwise comparisons to weigh competing priorities; and (6) My Plan—a tailored safety plan populated based on data supplied by the user in the previous sections. These components were followed by supplemental information: About Violence, Harmful Beliefs, and relevant Resources.

Follow-up data collection occurred 3 months later. After participants gave their informed consent, data collection procedures were identical to those at baseline. The myPlan Kenya app was offered to control participants after data collection.

### Analytic Sample

The DA was only assessed for intervention participants (N=177 at baseline; n=157 at 3 months [i.e., excludes those who were lost-to-follow-up]), as measurement was embedded within the myPlan Kenya app itself. To optimize the DA to the Nairobi context, a complete case approach for the outcome items was necessary, leading to a final sample size of 103 intervention participants who completed both baseline and follow-up; no significant differences were seen in the demographic characteristics of those retained versus lost-to-follow-up. No further missing data were observed.

### Measures

#### Primary Exposure: Baseline Risk for Severe or Lethal Violence (Danger Assessment Scale)

Risk for severe or lethal violence was measured at baseline among intervention participants only using the full 20-item DA Scale^10^ as developed and validated in the United States, including the aforementioned wording modifications for the Nairobi context (α Nairobi=0.84). This measure uses behavioral assessment to align with best practices for research on sensitive topics.^25^^,^^26^

#### Primary Outcome: Severe Intimate Partner Violence at 3-Month Follow-Up

IPV severity was assessed at 3-month follow-up using the 10-item Revised Conflicts and Tactics Scale (CTS-2; α=0.87).^27^ For each behavior, women were asked about the frequency of occurrence in the past 3 months: never (0), 1–2 times (1), 3+ times (2). A dichotomous variable was created indicative of severe violence, defined as a report of kicking and/or choking (CTS-2 items 5 and 6) 3 or more times in the past 3 months.

### Analysis

Characteristics of the analytic sample were first examined, overall and by IPV severity (less/more severe) at follow-up; chi-squared tests (binary/categorical variables), Fisher’s exact tests (categorical variables with cells<=5), and t-tests (continuous variables) assessed potential confounding by sample characteristics, with *P*<.1 used as the statistical significance threshold for confounding assessment. The prevalence of individual DA assessment items at baseline was then calculated by IPV severity at follow-up, with chi-squared tests assessing differences in baseline danger by follow-up IPV; Fisher’s exact tests were used for cells n<=5. The original DA score was created via summing responses to all 20 items (i.e., nonweighted because the original weighting was based on a U.S.-based study^10^). Phi and relative risk ratios (RRR) were calculated to determine items to include in the DA-Kenya. All items were retained in the DA-Kenya if they had an RRR greater than or equal to 2.0, except for item 13 (“Does he control most or all of your daily activities? For instance: does he tell you who you can be friends with, when you can see your family, how much money you can spend, whether or not you can use birth control?”), which was included based on previous literature. A weighted Kenya-DA was also created; due to small sample size, weighting was based on effect size. Specifically, RRR of 2 to <3=weight of 1; RRR of 3 to <4=weight of 2; RRR of 4 to <5=weight of 3; RRR of 5+=weight of 4. Diagnostic statistics, including C-statistics, sensitivity, and specificity, were examined for the original DA, Kenya-DA, and Kenya-DA weighted. Logistic regressions quantified odds of each metric predicting severe IPV at follow-up. The method suggested by Delong was used to test if the area under the curve (AUC) was significantly different (*P*<.05) between models.^28^ Statistical significance was set at *P*=.05.

### Ethical Approval

All procedures were approved by the Johns Hopkins Bloomberg School of Public Health Institutional Review Board and the Kenya National Commission for Science Technology. The trial was registered with the Pan African Clinical Trial Registry (PACTR201804003321122).

## RESULTS

### Summary of Formative Phase Danger Assessment Tailoring

Based on mock-ups from the formative phase, which was not specifically undertaken for the purpose of DA tailoring, as well as study team input, 2 DA items were modified to increase interpretation within and relevance for the Nairobi context. Specifically, the firearm item wording changed from “Has he ever used a weapon against you or threatened you with a lethal weapon?” and “If yes, was that weapon a gun?” to specify “machete, gun, sword, club, knife, whip, etc.” to reflect the range of lethal weapons a partner could use, rather than only guns. The illegal drug item was similarly adapted from “Does he use illegal drugs? By drugs, I mean ‘uppers’ or amphetamines, ‘meth,’ speed, angel dust, cocaine, ‘crack,’ street drugs, or mixtures.” to include local names for commonly used drugs: “Does he use drugs such as bhang, khat, heroin, or cocaine?” The pilot phase further revealed that at completion, the messages to women about their DA needed to be altered to reflect the high-danger context. Thus, rather than providing a message of “imminent risk” of severe violence, this wording was adapted to “increased risk” as the vast majority of women in the study were at the highest risk of severe violence.

The pilot phase further revealed that at completion, the messages to women about their DA needed to be altered to reflect the high-danger context.

### Characteristics of Intervention Participants at Baseline

Baseline characteristics of participants overall and by violence severity at follow-up are presented in Table 1. Just under half of the participants (46.6%) were enrolled at the Korogocho study site, with the remaining participants split between the Dandora and Huruma sites. IPV survivors were, on average, aged 27 years, and the majority identified as Kikuyu (39.8%) and Christian (91.3%). Most survivors were currently married (86.4%) and lived with their current partner (90.3%), though concurrent partnerships were relatively common (57.3% among male partners and 10.7% among participants). The majority of participants had completed less than primary education (60.2%) and were currently unemployed (92.2%).

**TABLE 1.** Sample Characteristics of myPlan Kenya Intervention Participants at Baseline by Violence Severity at Follow-Up, Nairobi, Kenya

	**Overall, No. (%)** **(n=103)**	**Follow-Up Violence Severity (n=103)**		
		**Less Severe IPV, % (n=61)**	**Severe IPV, % (n=42)**	***P* Value^a^**
Study site				
Korogocho	48 (46.6)	55.7	33.3	.08^b^
Dandora	32 (31.1)	24.6	40.5	
Huruma	23 (22.3)	19.7	26.2	
Age, mean (SD), years^c^	27.1 (4.7)	27.3 (5.0)	26.8 (4.3)	.61
Ethnicity				
Kikuyu	41 (39.8)	39.3	40.5	.63
Luo	30 (29.1)	31.2	26.2	
Luhya	13 (12.6)	14.8	9.5	
Other	19 (18.5)	14.8	23.8	
Religion				
Christian	94 (91.3)	95.1	85.7	.15
Other	9 (8.7)	4.9	14.3	
Recently migrated to Nairobi	25 (24.3)	23.0	26.2	.71
Current relationship status				
Married	89 (86.4)	82.0	92.9	.15
Unmarried	14 (13.6)	18.0	7.1	
Lives with current partner	93 (90.3)	85.3	97.6	.05^b^
Number of children, mean (SD)^c^	2.2 (1.2)	2.3 (1.2)	2.0 (1.1)	.27
Highest level of education completed				
Primary or less	62 (60.2)	60.7	59.5	.40
Some secondary	19 (18.5)	14.8	23.8	
Secondary or higher	22 (21.4)	24.6	16.7	
Currently unemployed	95 (92.2)	91.8	92.9	1.00
Concurrent partner (participant)	11 (10.7)	6.6	16.7	.12
Concurrent partner (partner)	59 (57.3)	57.4	57.1	.95

Abbreviation: IPV, intimate partner violence; SD, standard deviation.

^a^ T-tests used to test differences between violence severity for continuous variables. Chi-squared tests used to test differences between violence severity for binary/categorical variables; Fisher’s exact test for cells <5.

^b^ *P*<.1 for confounding assessment.

^c^ Continuous variable.

Significant differences in violence severity at follow-up were seen by study site (*P*=.08), where a larger proportion of participants from Dandora reported severe IPV than the other 2 sites (40.5% in Dandora vs. 33.3% in Korogocho vs. 26.2% in Huruma). A significantly higher proportion of those living with their current partner reported severe IPV at follow-up (*P*=.05).

### Danger Assessment Items and Associations With Severe Violence at 3-Month Follow-Up

Prevalence of DA items at baseline are presented in Table 2. The DA items that were most prevalent included: partner being violently or constantly jealous (89.3%), having left at least temporarily during the past year (82.5%), forced sex (82.5%), partner following or spying (75.7%), and controlling most or all of daily activities (73.8%).

**TABLE 2.** Prevalence of Danger Assessment Items at Baseline and Correlation With Violence Severity at Follow-Up Among myPlan Kenya Intervention Participants, Nairobi, Kenya

**Item**	**Overall Item Prevalence at Baseline, %**	**Item Prevalence at Baseline by Follow-Up**^a^ **Violence Severity** **(n=103)**			**Phi**	**RRR** **(95% CI)**	**Item** **Weight**^b^
		**Less Severe IPV, % (n=61)**	**Severe IPV, % (n=42)**	***P* Value**			
1. Has the physical violence increased in severity or frequency over the past year?	75.7	68.9	85.7	.05	0.19	2.71 (0.98, 7.52)	1
2. Does he own a dangerous weapon he would use during a fight (machete, gun, sword, club, knife, whip, etc.)?	62.1	49.2	81.0	.001	0.32	4.39^c^ (1.75, 11.01)	3
3. During the past year, have you left him after living together or being a couple (even if you got back together)?	82.5	80.3	85.7	.48	0.07	1.47 (0.50, 4.28)	not retained
4. Is he unemployed?	37.9	31.2	47.6	.09	0.17	2.01 (0.89, 4.52)	1
5. Has he ever used a weapon against you or threatened you with a lethal weapon (machete, gun, sword, club, knife, whip, etc.)?	66.0	55.7	81.0	.008	0.26	3.38^c^ (1.34, 8.48)	2
6. Does he threaten to kill you?	64.1	55.7	76.2	.03	0.21	2.54^d^ (1.06, 6.07)	1
7. Has he previously been in trouble with the police or chief for violence against his partner or children?	52.4	44.3	64.3	.05	0.20	2.27^d^ (1.01, 5.09)	1
8. Do you have a child that is not his?	32.0	36.1	26.2	.29	0.10	0.63 (0.27, 1.49)	not retained
9. Has he ever forced you to have sex when you did not wish to do so?	82.5	73.8	95.2	.007	0.28	7.11^c^ (1.53, 32.35)	4
10. Does he ever try to choke you?	58.3	39.3	85.7	<.001	0.46	9.25^c^ (3.38, 25.28)	4
11. Does he use drugs, such as bhang, khat, heroine, or cocaine?	49.5	44.3	57.1	.20	0.13	1.67 (0.76, 3.71)	not retained
12. Does he have a drinking problem?	55.3	52.5	59.5	.48	0.07	1.33 (0.60, 2.95)	not retained
13. Does he control most or all of your daily activities? For instance: does he tell you who you can be friends with, when you can see your family, how much money you can spend, whether or not you can use birth control?	73.8	68.9	81.0	.17	0.14	1.92 (0.75, 4.93)	1
14. Is he violently and constantly jealous of you? (For instance, does he say “If I can't have you, no one can”?)	89.3	83.6	97.6	.03	0.22	8.04^d^ (0.99, 65.40)	4
15. Have you ever been beaten by him while you were pregnant?	61.2	49.2	78.6	.003	0.30	3.79^c^ (1.55, 9.24)	2
16. Has he ever threatened or tried to commit suicide?	32.0	23.0	45.2	.02	0.23	2.77^d^ (1.18, 6.50)	1
17. Does he threaten to harm your children?	19.4	9.8	33.3	.003	0.29	4.58^c^ (1.58, 13.2)	3
18. Do you believe he is capable of killing you?	58.3	49.2	71.4	.02	0.22	2.58^d^ (1.11, 5.96)	1
19. Does he follow or spy on you, leave threatening notes or messages, destroy your belongings, or call you when you don't want him to?	75.7	68.9	85.7	.05	0.19	2.71 (0.98, 7.53)	1
20. Have YOU ever threatened or tried to commit suicide?	33.0	26.2	42.9	.08	0.17	2.11 (0.91, 4.86)	1

Abbreviations: CI, confidence interval; IPV, intimate partner violence; RRR, relative risk ratio.

^a^ Chi-squared tests used to test difference in baseline danger assessment item between violence severity at follow-up; Fisher’s exact test for cells <5.

^b^ Due to small n’s, weighting for Kenya-Danger Assessment weighted was based on effect size rather than statistical significance. RRR of 2 to <3=weight of 1; RRR of 3 to <4=weight of 2; RRR of 4 to <5=weight of 3; RRR of 5+=weight of 4.

^c^ *P*<.01.

^d^ *P*<.05.

Table 2 further presents the degree of association between baseline DA items and follow-up violence severity via Chi-squared statistics, Phi, and RRRs. Almost all of the items displayed positive associations with follow-up violence severity, with the exception of item 8, where having a child that was not the partner’s displayed a nonsignificant inverse association with severe IPV at follow-up.

Strongest associations between baseline DA risk factors and follow-up violence severity were seen for the following items: “Does he ever try to choke you?” (RRR: 9.25; 95% confidence interval [CI]=3.38, 25.28; *P*<.01); “Is he violently and constantly jealous of you? (For instance, does he say, “If I can’t have you, no one can”?)” (RRR: 8.04; 95% CI=0.99, 65.40; *P*<.05); and “Has he ever forced you to have sex when you did not wish to do so?” (RRR: 7.11; 95% CI=1.53, 32.35; *P*<.01); these 3 items were given highest weighting for subsequent weighted analyses. Additional items strongly associated with follow-up violence severity comprised: “Does he own a dangerous weapon he would use during a fight (machete, gun, sword, club, knife, whip, etc.)?” (RRR: 4.39; 95% CI=1.75,11.01; *P*<.01); “Has he ever used a weapon against you or threatened you with a lethal weapon (machete, gun, sword, club, knife, whip, etc.)?” (RRR: 3.38; 95% CI=1.34, 8.48; *P*<.01); “Have you ever been beaten by him while you were pregnant?” (RRR: 3.79; 95% CI=1.55, 9.24; *P*<.01); and “Does he threaten to harm your children?” (RRR: 4.58; 95% CI=1.58, 13.2; *P*<.01). Items with strongest associations (RRR>=2.0) were then retained for the 16-item Kenya-DA.

Diagnostic statistics comparing the 3 DA configurations (original DA, Kenya-DA, and Kenya-DA weighted) are displayed in Table 3 and the Figure. Means ranged based on configurations: original DA (mean=11.6; range=0–20); Kenya-DA (mean=9.0; range=0–6); Kenya-DA weighted (mean=19.2; range=0–31). Specificity was highest for the Kenya-DA weighted (77.05%) followed by the original DA (75.41%); however, sensitivity was lowest for the original DA (57.14%), resulting in the overall lowest C-statistic. In comparing the Kenya-DA weighted to the Kenya-DA, the Kenya-DA weighted produced slightly higher sensitivity (66.67% vs. 64.29%) and specificity (77.05% and 72.13%), resulting in the highest C-statistic (0.78 vs. 0.75). All 3 configurations were significantly predictive of severe IPV at follow-up (original DA: OR=1.26; 95% CI=1.12, 1.41; *P*<.001; Kenya-DA: OR=1.33; 95% CI=1.16, 1.53; *P*<.001; Kenya-DA weighted: OR=1.19; 95% CI=1.10, 1.28; *P*<.001).

**TABLE 3.** Diagnostic Statistics and Logistic Regressions on Responses to the Nairobi Danger Assessment Scale for Risk of Severe IPV Among IPV Survivors in Nairobi, Kenya

	**C-** **Statistic**	**Sensitivity, % (n=103)**	**Specificity, % (n=103)**	**OR (95% CI)**
Original Danger Assessment	0.73	57.14	75.41	1.26 (1.12, 1.41)^a^
Kenya-Danger Assessment	0.75	64.29	72.13	1.33 (1.16, 1.53)^a^
Kenya-Danger Assessment Weighted	0.78	66.67	77.05	1.19 (1.10, 1.28)^a^

Abbreviations: CI, confidence interval; IPV, intimate partner violence; OR, odds ratio.

^a^ *P*<.001.

**FIGURE:**
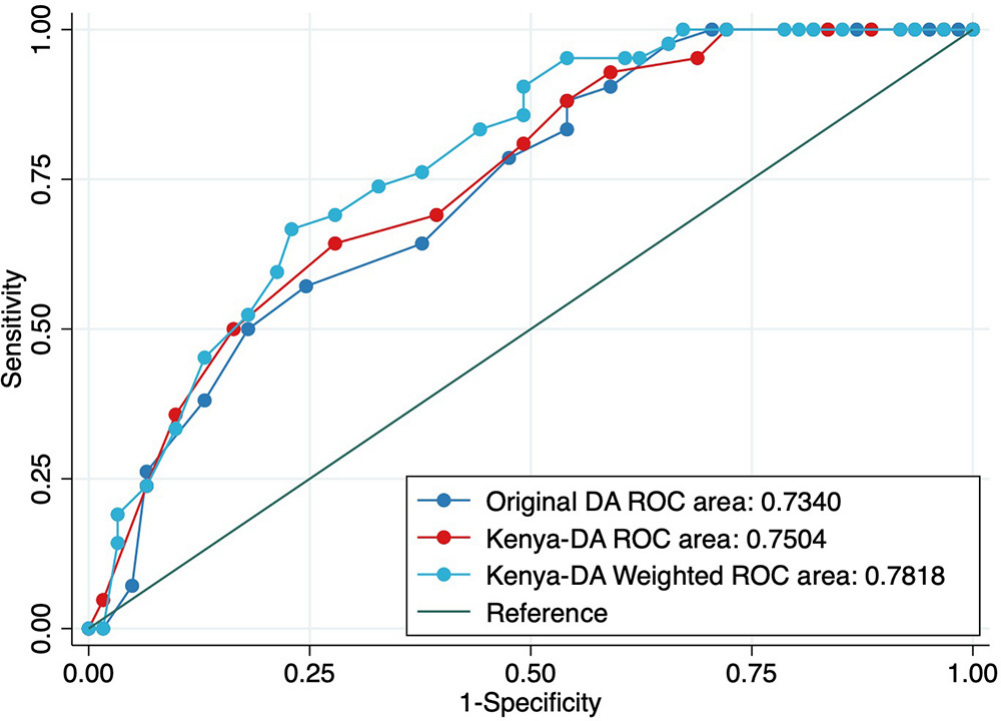
ROC Curves Comparing Diagnostic Statistics on Responses to Original DA, Kenya-DA, and Kenya-DA Weighted Among Intimate Partner Violence Survivors, Nairobi, Kenya Abbreviation: DA, Danger Assessment; ROC, receiver operating characteristic.

Receiver operating characteristic (ROC) curves comparing the original DA, Kenya-DA, and Kenya-DA weighted are presented in the Figure. The greatest AUC is observed for the Kenya-DA weighted (0.78) followed by the Kenya-DA (0.75); the original DA produced the lowest ROC estimates (0.73). The chi-squared test found that the Kenya-DA fit significantly better than the original DA (*P*=.01), and the Kenya-DA weighted fit significantly better than the Kenya-DA (*P*=.02).

## DISCUSSION

This first use of the DA within a high-danger, low- and middle-income country context found that the DA performed well within this population, with all configurations (original DA, Kenya-DA, and Kenya-DA weighted) producing AUC estimates ranging from 0.73 to 0.78. Although previous U.S.-based research assessing the validity and reliability of the DA has estimated a higher AUC (0.86), diagnostics for the DA overall remain markedly higher than other similar violence risk assessments.^7^ Minor discrepancies in diagnostic criteria across configurations reflect the utility of this tool. Namely, the Kenya-DA weighted produced the highest AUC, sensitivity, and specificity estimates, though increased diagnostic capacity was minor compared to the Kenya-DA. Prediction of severe IPV across configurations could not be compared, given differences in scales. In line with calls to translate these valuable assessment tools from violence research into practice,^6^^–^^8^ we ultimately recommend both the Kenya-DA and Kenya-DA weighted. The Kenya-DA may be best suited for prevention and response practitioners working directly with violence survivors due to the ease of tool implementation, brevity, and straightforward scoring. The Kenya-DA weighted can add additional accuracy in research settings but may be more difficult to score and interpret.

This first use of the DA within a high-danger low- and middle-income country context found that the DA performed well within this population.

An understanding of the risk of severe violence by an abusive partner in the context of informal settlements in Nairobi and how specific behaviors by the partner increase risk and predict severe violence is similarly valuable for direct service providers. Notably, women’s reports of being choked and having a violent and constantly jealous partner were the 2 strongest predictors of severe IPV at 3-month follow-up (aRRR=9.25; 95% CI=3.38, 25.28; *P*<.01 and aRRR=8.04; 95% CI=0.99, 65.40; *P*<.05, respectively). Moreover, violently and constantly jealous behaviors were the most commonly reported experiences, with approximately 89% of IPV survivors reporting these behaviors from their partner in the past 3 months. Given the high frequency of this extreme jealous behavior, health care providers and direct service providers should include violently and constantly jealous behaviors as part of standard conversations with clients to better understand the severity of these behaviors and identify women who are at increased risk of severe violence. Most abusers are jealous; however, this extreme jealousy may serve as an important predictor of more severe violence and, in future research, could be asked about during a brief screening without implementation of the entire 16 items on the DA or other risk assessments.

The third item given the highest weighting for the Kenya-DA weighted, with an RRR of 7.11 (95% CI=1.53, 32.35; *P*<.01), was forced sex, indicating that sexual violence is an important risk factor of violence severity within an abusive intimate partnership. In Nairobi, like urban areas globally, there has been a focus by authorities on preventing and responding to nonpartner sexual violence and assault (i.e., “stranger danger”). The study findings speak to what women have known—that forced sex is not only perpetrated by strangers but that women are experiencing sexual violence in their intimate relationships and that sexual violence is a risk factor for women’s experience of severe violence. Kenya Demographic and Health Survey data indicate that 10% of partnered women experienced sexual violence from their spouse within the past year, and among ever-partnered women, experiences of sexual violence were primarily perpetrated by a current or former intimate partner.^29^ Health care providers, in particular, must ensure that sexual violence screenings are inclusive of spousal/partner sexual violence to provide survivors with the sexual, reproductive, and psychosocial resources needed to mitigate the health, economic, and social impact of this severe form of violence. At the macro-level, prevention programs and legislation are needed that target changes in social norms—norms that minimize the seriousness and negative impact of sexual violence within intimate partnerships and inhibit women from seeking help because of shame and stigma.

Some findings on the prevalence of risk factors at baseline and their associations with severe violence at follow-up were surprising. Foremost, partner drug use (57.1%, severe IPV; 44.3%, no severe IPV) and heavy alcohol use (59.5%, severe IPV; 52.5%, no severe IPV) were both common among women who reported and those who did not report severe violence at follow-up. However, neither of these factors was predictive of severe violence; both factors have been found to be strongly associated with risk of IPV repeat assault in the United States,^15^ though to a lesser extent for intimate partner homicide^10^ and among immigrant samples.^16^ Moreover, although male unemployment ranged from one-third to nearly half of partners among the 2 groups (47.6%, severe IPV; 31.2%, no severe IPV), it was not as high as expected. In contrast to results from the United States and other settings,^14^ male unemployment was **not** significantly predictive of severe violence. Notably, these data were collected before the COVID-19 pandemic (2018). The COVID-19 pandemic and related economic fallout may have not only increased unemployment for women and their partners but also heightened the risk of severe violence for women in already fragile economic settings. Though not significantly associated with severe IPV, male partner unemployment was ultimately retained within the Kenya-DA/Kenya-DA weighted because the RRR was more than 2 (2.01; 95% CI=0.89, 4.52). Future research should continue to monitor the impact of economic insecurity on IPV in Nairobi and similar contexts.

These results hold promising programmatic implications for service providers and Kenyan women themselves. In other settings, such as the United States, the DA has had a high practical value in enabling rapid assessment of risk for severe or lethal violence and linking women to first responders, including police and emergency personnel.^8^ Now, with a validated Kenya-DA, a similar approach of training and equipping direct service providers (e.g., health/psychosocial, social service, justice/chiefs, housing, faith-based, and educational) can result in an increase in earlier identification of women in dangerous intimate relationships. For direct care providers, administration of the DA could occur as part of routine visits, completed while waiting to see the care provider, either independently or with confidential support within a private setting. The results could then be reviewed as part of the visit, and safety plans could be developed to fit women’s individual needs and situations. Providers could also directly refer women to other violence support services, including health, justice, and social services.

Now, with a validated Kenya-DA, a similar approach of training and equipping direct service providers can result in an increase in earlier identification of women in dangerous intimate relationships.

Initially, the DA was designed to be a collaborative tool used between violence, health, and/or justice responders and IPV survivors.^30^ However, given the vast mobile infrastructure in Kenya, this tool could additionally be useful for women to undertake via mobile phone on their own or in more informal settings, with linkage to further care as needed. Although the formal violence response service infrastructure within Nairobi and across Kenya is rapidly expanding, the majority of women still seek support from informal sources (e.g., family, neighbors, and friends).^29^ As such, the Kenya-DA is a brief and easy-to-use tool for informal support in their discussions with friends/family members experiencing IPV. Tools such as myPlan Kenya^31^ can provide assessment of homicide risk as well as safety planning and linkage to violence support services. Further, as in the United States and beyond, the Kenya-DA can be used by women to assess their own situation, understand the danger in their relationship, and make a safety plan that includes reaching out for help from informal and formal services.

### Limitations

The present research should be considered in light of several limitations. Namely, the gold standard for assessing risk factors to predict severe violence necessitates a longitudinal study and has multiple ethical challenges. Therefore, the present methodology offers a promising solution for validation of a risk assessment tool while addressing ethical and safety considerations. Severity of violence does not measure homicide but serves as a proxy for more lethal violence (i.e., murder of women by a partner or ex-partner). Moreover, the wording of some items is similar for both assessments of “danger” and “severe violence” (i.e., Has he ever forced you to have sex when you did not wish to do so?”); although such items would be conflating exposure and outcome within a cross-sectional design, concerns have been minimized given longitudinal data. Lastly, the present secondary analysis was limited to intervention participants within a trial for a safety decision aid, and, therefore, sample size was suboptimal for some analyses, including our ability to stratify by sociodemographic characteristics. Limitations surrounding event rate and sample size further contributed to large confidence intervals; future research should aim to overcome these limitations to examine both effect size and statistical significance in weighting cut-offs. Generalizability is limited to urban informal settlements of Nairobi, and further research is needed to understand external validity within rural areas and other informal settlements globally. Future research aims to continue validation within larger samples to further our understanding of safety risks for women in Kenya, as well as explore dissemination and implementation avenues to ensure alignment of research with practice.

## CONCLUSION

The Kenya-DA and Kenya-DA weighted are valuable tools for practice and research. The Kenya-DA weighted, while slightly more complex to score, can increase accuracy in risk assessments for severe violence and homicide—this tool is valuable for research organizations ready to undertake such analyses. The global burden of premature death of women at the hands of current and ex-partners is extremely high and thus demands a rapid and effective response to prevent further death. The validated Kenya-DA and Kenya-DA weighted are important resources for survivors, advocates, health providers, and researchers to be integrated into an evidence-based and comprehensive approach to IPV prevention and response globally.

## Notes

**Peer Reviewed**

**First published online:** January 31, 2024.
